# A simple, biologically sound, and potentially useful working classification of Chagas disease vectors

**DOI:** 10.1590/0074-02760160203

**Published:** 2016-10

**Authors:** Fernando Abad-Franch

**Affiliations:** Fundação Oswaldo Cruz, Centro de Pesquisas René Rachou, Belo Horizonte, MG, Brasil

**Keywords:** Triatominae, hierarchical classifications, Chagas disease

## Abstract

Current working classifications of Chagas disease vectors rely on a loose mix-up of biological and operational matters. They are therefore confusing and ineffective. I propose a very simple classification that makes biological sense and can be operationally useful. It considers a four-level hierarchy of *species* (which can be native or non-native); *populations* (either wild or non-wild); infestation *foci* (natural, domestic or peridomestic); and *individual bugs* (which can be solitary house-invaders or part of a hidden infestation focus). This classification translates into a clear, algorithmic scheme for triatomine control-surveillance that may be useful at every operationally relevant scale, from multi-country initiatives to on-site control-surveillance action.

Triatomine bugs feed on vertebrate blood. As a side-effect of this habit, they transmit *Trypanosoma cruzi*, the parasite that causes Chagas disease. Chagas disease is a major public health concern in the Americas, and vector control remains, together with blood- and organ-donor screening, the cornerstone of primary disease prevention (Rassi et al. 2010). With over 140 known triatomine species, each with its own lifestyle and epidemiological relevance, a working classification of these vectors seems warranted. Current working classifications of triatomines, however, rely on a rather loose mix-up of biological and operational matters. This makes them confusing and, hence, potentially ineffective. Here I propose a very simple classification scheme that may be useful over the spatial and operational scales relevant to triatomine control-surveillance and that, at the same time, makes biological sense.

Not all triatomines are born equal. Biologically, each individual belongs to a family of siblings (often clustered together in an ‘infestation focus’) nested within a population nested within a species. Higher groupings, such as species groups or ‘complexes’, genera, or tribes, have only historical meaning - if correctly defined, they roughly trace the common ancestry of lower-level groupings. From a public health perspective, some triatomines are more dangerous than others, and this mainly depends on how they interact with us humans; operationally, in addition, some bugs are easier to control than others. We would like to have a tidy working classification that adequately captures these crucial biological and operational matters - but do we?

The most popular working classifications to date have been those distinguishing ‘primary’ from ‘secondary’ vectors or ‘domestic’ from ‘sylvatic’ triatomines - occasionally with further subsets such as ‘candidate vectors’ or ‘intrusive’ *vs*. ‘domiciliary’ *vs*. ‘domestic’ species (*e.g.*, Noireau et al. 2005, Noireau & Dujardin 2010). As a recent review shows, triatomine species or populations have overall been sorted into at least 15 categories and sub-categories ranging from ‘domestic’ or ‘domesticated’ to ‘essentially sylvatic’ (see Table II of Waleckx et al. 2015). Arguably, real-life vector control-surveillance programs would benefit from simpler, yet sound, working classifications.

One potential approach to clarifying the tangled landscape of biological-operational classifications is to get hierarchical about the problem. In fact, the biological hierarchy of triatomine individuals, infestation foci, populations, and species is mirrored by the operational hierarchy of field (on-site) control-surveillance interventions, local-level control programs, national programs, and international initiatives. I build on this idea to develop a working classification that is fairly clear and tidy and, I believe, can become operationally useful while making full biological sense - also in that it dispenses with “...the teleological and anthropocentric concept that triatomines are evolving towards greater adaptation to the domestic environment...” (Barrett 1991). This working classification considers four hierarchical levels.

*Level one: species -* I first note, with some emphasis, that there are no ‘domestic’ triatomine species: whether we know much, little, or almost nothing about them, all triatomine species obviously encompass sylvatic or wild populations across their natural ranges. It is prudent to assume, moreover, that bugs of virtually any species can, at least on occasion, infest a house. When talking of species, then, the ‘sylvatic *vs*. domestic’ dichotomy is false and we should stop using it. However, a species can be either ‘native’ or ‘non-native’ to a given territory. This species-level dichotomy is both true and of great practical importance: non-native (introduced) triatomine species occur only in man-made habitats and can hence, in principle, be eliminated, whereas native species cannot. This, in turn, is critical for addressing the broad-scale issues that international initiatives care about - *i.e.*, the planning, co-ordination and evaluation of national and multinational vector control programs (Figure). Importantly, there are just a few relevant examples of non-native triatomine species in the Americas - *Rhodnius prolixus* out of the Orinoco basin, *Triatoma infestans* out of the Chaco and parts of the central-southern Andes, and *T. dimidiata* in coastal Ecuador and north-western Peru. Most of these non-native bugs have effectively been controlled or eliminated, yet much remains to be done before *R. prolixus* and *T. dimidiata* are eliminated from, respectively, northern Colombia and western Ecuador. *T. rubrofasciata* is most likely non-native to the Americas but is considered, as yet, of lesser epidemiological importance in the region.

**Figure:**
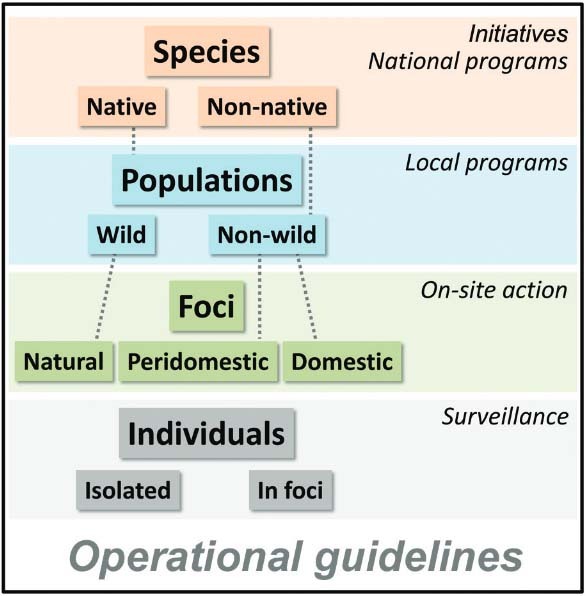
A four-level hierarchical classification of triatomines and its relationship with hierarchically-structured vector control-surveillance strategies (in italics); the outer black rectangle stresses the crucial, overarching role of local operational guidelines.

*Level two: populations* - Local populations of a given native species may or may not colonise in man-made structures. We can thus have ‘wild’ and ‘non-wild’ populations of a native species. This is relevant for local (municipal, state/province/department) control-surveillance planning and evaluation. For example, there are non-wild *R. ecuadoriensis* populations in some areas of western and south-western Ecuador, as well as in north-western Peru, but only wild populations have so far been found in the wet premontane forests of the central-western slope of the Ecuadorian Andes. Control-surveillance planning and evaluation must take this into account at the local level (Figure).

*Level three: foci* - A non-wild triatomine population can spawn infestation foci inside or around houses - that is, ‘domestic’ or ‘peridomestic’ breeding foci. Wild populations consist of ‘natural’ foci in non-man-made microhabitats. This distinction is relevant at the frontline, where control-surveillance agents deploy on-site control interventions (Figure). As a control-surveillance field team visits a dwelling, the agents must search for infestation foci and determine a course of action depending on the characteristics of those foci (location and size, bug species, infection with *T. cruzi*, etc.) and operational guidelines (sometimes, for example, it may be wise to assume that peridomestic foci of species known to colonise inside houses do imply domestic foci, which may be more difficult to detect; see Abad-Franch et al. 2014a).

*Level four: individuals* - Finally, adult (or, rarely, immature) individual bugs of virtually any species (native or non-native) or population (wild or non-wild) can enter a house (by flying, by passive carriage, or by walking from a nearby infestation focus) without establishing a new breeding focus. This is relevant for surveillance, including early disease case-detection; importantly, the recording and reporting of house-invasion events can indicate whether entomological surveillance is working adequately even in areas without domestic/peridomestic infestation foci (Figure).

A (simplified) vector control-surveillance algorithm applying these ideas would roughly go like this.

(1) - Is there any non-native triatomine *species* in your region or country? If so, develop highly co-ordinated, area-wide insecticide-spraying campaigns to eliminate it - that is, seek inspiration in the successful initiatives against non-native *T. infestans* (Southern Cone) and *R. prolixus* (Central America). Irrespective of whether non-native species occur in your region or country, go to point 2. (And, if in doubt about the native/non-native status of any species in your region or country, prioritise research aimed at quickly settling the issue).

(2) - Are there any non-wild *populations* of any native species anywhere in the area under your administration? If so, develop a long-term, carefully designed program for the detection and elimination of domestic and peridomestic infestation foci wherever such populations are recorded (see point 3); run this program indefinitely (see Abad-Franch et al. 2013, 2014b). Otherwise, keep entomological and epidemiological surveillance active - and especially so if any native species in your area is known to include non-wild populations elsewhere (for example, non-wild *Panstrongylus megistus* populations do not seem to occur in southern Brazil, but they do in other areas of the country).

(3) - Are there any domestic or peridomestic infestation *foci* in the dwelling you are visiting as part of a control-surveillance activity? If so, eliminate those foci and take any additional action prescribed by your operational guidelines (*e.g.*, collect bugs and draw blood samples from dwellers to test for infection, etc.). Otherwise, explain to dwellers how important their active involvement in entomological surveillance is and go to the next dwelling in your working schedule.

(4) - Finally, have you recorded the presence of any *individual* triatomine (usually an adult) inside a house? If so, determine whether it is an isolated individual or part of an infestation focus (*i.e*., go back to point 3); if the bug is a lone invader, follow your operational guidelines (*e.g.*, test the bug, and possibly the dwellers, for infection, deliver information on the disease and its vectors, etc.) and consider that it may be useful to also look for natural foci (*e.g.*, in palms or rocky outcrops) near the house. If not (*e.g.*, dwellers reported a non-triatomine reduviid), stimulate dwellers to keep involved in community-based entomological surveillance.

Simple rules like these ones can, I believe, help sustain effective Chagas disease control in the long run. It is nonetheless clear that, in developing a very general and flexible proposal, I have ignored or barely touched upon many important details - chiefly those bearing on vectorial capacity. These details can easily, and must, be incorporated into *local operational guidelines* (Figure) - in which, for example, triatomine taxonomy (particularly for native species), house infestation metrics, or *T. cruzi* infection rates (particularly for non-wild populations) will often steer decision-making. When combined with sound operational guidelines, the working classification I have presented has potential to substantially strengthen the long-term prevention of vector-borne Chagas disease.
